# Predicting Characteristics of Dissimilar Laser Welded Polymeric Joints Using a Multi-Layer Perceptrons Model Coupled with Archimedes Optimizer

**DOI:** 10.3390/polym15010233

**Published:** 2023-01-02

**Authors:** Essam B. Moustafa, Ammar Elsheikh

**Affiliations:** 1 Mechanical Engineering Department, Faculty of Engineering, King Abdulaziz University, Jeddah 21589, Saudi Arabia; 2 Department of Production Engineering and Mechanical Design, Faculty of Engineering, Tanta University, Tanta 31527, Egypt

**Keywords:** laser welding, polymeric lap joints, artificial intelligence, multi-layer perceptrons, Archimedes optimizer

## Abstract

This study investigates the application of a coupled multi-layer perceptrons (MLP) model with Archimedes optimizer (AO) to predict characteristics of dissimilar lap joints made of polymethyl methacrylate (PMMA) and polycarbonate (PC). The joints were welded using the laser transmission welding (LTW) technique equipped with a beam wobbling feature. The inputs of the models were laser power, welding speed, pulse frequency, wobble frequency, and wobble width; whereas, the outputs were seam width and shear strength of the joint. The Archimedes optimizer was employed to obtain the optimal internal parameters of the multi-layer perceptrons. In addition to the Archimedes optimizer, the conventional gradient descent technique, as well as the particle swarm optimizer (PSO), was employed as internal optimizers of the multi-layer perceptrons model. The prediction accuracy of the three models was compared using different error measures. The AO-MLP outperformed the other two models. The computed root mean square errors of the MLP, PSO-MLP, and AO-MLP models are (39.798, 19.909, and 2.283) and (0.153, 0.084, and 0.0321) for shear strength and seam width, respectively.

## 1. Introduction

Polymeric materials have been widely used in many engineering, domestic, and medical applications, including electronics, automotive, aerospace, medical, implants, medical disposals tissue engineering, dental materials, wind turbine, solar cells, packaging, and sensors [1,2,3,4,5]. This is due to the advantages over other metallic materials such as being lightweight, having good specific strength, and being a cost-efficient processing technique. Joining polymeric materials is a critical industrial issue to obtain high-quality products that fit a range of industrial and daily-life applications [6,7,8]. Many joining techniques of polymeric materials have been developed in literature such as hot plate welding [9], hot gas implant welding [10], hot gas butt welding [11], friction stir welding [12,13], electromagnetic pulse welding [14], vibration welding [15], and ultrasonic welding [16,17]. Laser welding has shown promising applications in the welding of polymeric materials due to its flexibility, versatility, and speed, producing narrow and deep welds, non-continuous nature, and non-contact nature [18,19]. There are two widespread laser welding technologies used in polymeric material welding, namely, laser transmission welding (LTW) and laser butt welding (LBW). In LBW, the ends of the welded parts are placed together to form a butt joint and the laser beam irradiates the side surfaces of the two parts. The laser energy is absorbed by the two welded parts and converted into heat, which is utilized to convert the welding interface into a softened state. Once the welding interface is converted into a softened state, they are joined together by applying pressing forces on the welded parts [20]. In LTW, one of the welded parts is put on the other one and the laser beam irradiates the upper surface of the upper part [21]. The laser beam is passed through the transparent upper part and absorbed by the lower part in which it is converted into heat. The upper transparent part may also absorb a small amount of laser energy due to the occurrence of multiple scattering. This heat, along with clamping forces, is utilized to accomplish the welding process with minimum energy input [22]. LTW has been extensively used to weld thin and thick polymeric components [23].

Many experimental studies have been carried out to explore LTW of polymeric materials by investigating the effects of optical and thermophysical properties of welded materials, applying coating on the welded materials, welding paraments and conditions, and laser beam characteristics on the performance of the LTW process [18,19,24]. The main welding paraments and conditions that affect the induced heat during the welding process are laser power, welding speed, pulse frequency, laser beam size, and clamping pressure. Chen et al. [25] investigated LTW of polymethyl methacrylate (PMMA) sheets by employing copper wires as an absorbing medium that absorbs the laser energy and converts it into heat. The shear force of welded joint using multi-core wire is higher than that of single-core wire by about 33.7–124.3% and the maximum obtained shear force was 1100 N. The reason behind this enhancement in the joint strength is that the melted PMMA material flowed into multi-core wire which resulted in a substantial increase in the Van der Waals force between PMMA and copper wires. Wang et al. [26] welded polyarylsulfone (PASF) using the LTW technique utilizing carbon black and zinc particles as absorbing mediums. The effects of welding parameters (welding speed and laser power) on the joint strength and interface structure were investigated. The welded joints have a maximum welding strength of 10.8 MPa at a welding speed of 3 mm/s and a laser power of 26 W. Irregular ridges and bubbles were found in the weld interface due to the existence of coating particles and confined gasses in the molten pool. Jankus and Bendikiene [27] investigated the effects of material transparency during LTW of polyphthalamide (PPA) sheets with carbon black additives on the weld interface properties. It was observed that more pores are formed on the weld interface as meltdown increases. The weld strength is highly affected by the formation of defects inside the weld seam. Yuet et al. [28] investigated LTW of dissimilar thermoplastics, namely polycarbonate (PC) and PASF. Metal particles, including tin, zinc, and magnesium zinc alloy, have been employed as absorbents to enhance the optical properties of welded materials. Experiments were conducted to explore the effects of welding parameters (welding speed and laser power) as well as metal particles on the weld morphologies and shear strength. The welded joint with magnesium zinc alloy particles had the highest shear strength among other particles. The shear force of these joints reached a high value of 662 N at a welding speed of 8 mm/s and laser power of 35 W. The shear force reached its peak values at moderate values of laser powers and welding speeds. Acherjee [29] modeled LTW of dissimilar welded joints made of PC and acrylonitrile butadiene styrene (ABS) using ANSYS^®^ as a powerful commercial finite element and parametric design software. All heat transfer modes were considered in the developed models including heat convection, conduction, and thermal radiation. The numerical results were confirmed by conduction welding experiments. Both experimental and simulated results were in good agreement. It was observed that the melt width at the weld interface is not the same in both welded materials because of the difference between the thermophysical properties of ABS and PC, especially the glass transition temperatures.

Prediction of the joint characteristics produced by LTW has significant importance in optimizing the joint quality. Artificial intelligence tools have been reported as efficient tools to model and predict the response of different engineering processes such as metal cutting [30], desalination systems [31], power generation plants [32], material processing [33], wastewater treatment plant [34], solar systems [35], and heat exchangers [36]. They also performed well in the modeling manufacturing process of polymeric materials such as friction stir welding [37], ultrasonic welding [38], and laser cutting of polymers [39].

AI tools were also employed to model the laser welding process of different materials and joint configurations [40,41,42]. Banerjee et al. [43] introduced an ANN model to predict the weld quality of stainless steel laser-welded butt joints. The model inputs were laser power, wire feed rate, and welding speed; whereas, the model outputs were overlap factor and tensile strength. The introduced model was trained using the Levenberg–Marquardt optimizer. The optimal number of hidden nodes was determined. The average percentage error between actual and predicted data was 1.22 and 1.86 for overlap factor and tensile strength, respectively. The shear force of dissimilar lap-welded joints made of copper and aluminum was predicted using the ANN technique [44]. A prediction accuracy of 91% was found between the predicted and experimental data. ANN models were developed to estimate weld geometry [45] and weld depth [46] of laser-welded joints made of titanium alloy and boron steel, respectively. Defects induced in laser-welded joints made of galvanized steel sheets were detected using the ANN model [47]. Conventional artificial tools suffer low accuracy due to the well-known drawbacks of the conventional optimizers embedded in the artificial intelligence models such as trapping at local minima, low convergence rates, and high computational costs [48]. Metaheuristic optimizers such as Harris Hawk’s Optimizer [49], mayfly optimizer [50], chimp optimizer [51], heap-based optimizer [52], transient search optimizer [53], pigeon optimizer [54], cat swarm optimizer [55], rabbit optimizer [56], and parasitism-predation algorithm [57] have been proposed as internal optimizers to optimize different artificial intelligence models. Datta et al. [58] employed four different metaheuristics optimizers genetic algorithm, grey wolf optimizer, Jaya optimizer, bonobo optimizer, and sine-cosine optimizer to optimize two different ANN models, namely feed-forward neural network and recurrent neural network, used to predict the characteristics of laser-welded butt joints made of nickel–titanium alloy. The inputs of the models were power, welding speed, duty factor, frequency, and focal distance. The outputs of the models were bead geometry, tensile strength, and microhardness. The performance of the ANN models was improved by employing metaheuristic optimizers. The best results were obtained by the bonobo optimizer followed by the genetic algorithm, grey wolf optimizer, and Jaya optimizer; whereas, the sine-cosine optimizer had the worst accuracy. Liu et al. [59] employed a genetic algorithm to optimize the ANN model used to predict the joint characteristics of laser-welded butt joints made of SUS316L stainless steel. The inputs of the model were power, welding speed, beam separation, and focal distance. The outputs of the models were the number of porosities and their average area. The genetic algorithm was also employed to optimize the welding process to obtain porosity-free welds. In another study, Yanxi et al. [60] applied optimized ANN using the genetic algorithm to predict the weld geometry of laser-welded joints. Wu et al. [61] developed a hybrid ANN/bees optimizer model to reduce the distortion of laser-welded joints.

Based on the discussed literature, AI tools succeeded to model different laser welding processes, predict joint characteristics, and detect the defects in the welded joints. Moreover, the integration between AI tools and metaheuristic optimizers outperformed standalone AI tools. This motivated us to develop a hybrid MLP integrated with AO to predict the seam width and shear strength of dissimilar laser-welded joints. AO was employed to improve the prediction accuracy of conventional MLP mode. An optimized MLP model using PSO as well as standalone MLP was employed to predict the welded joint characteristics. Different error measures were utilized to assess the performance of different models. 

## 2. Experimentation

**Figure 1:**
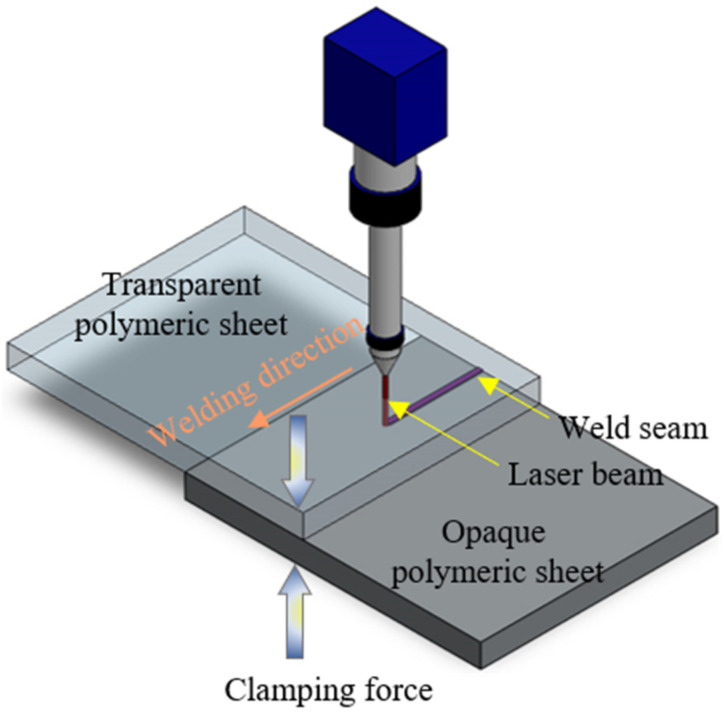
Experimental setup.

Transparent PMMA and PC sheets of the same size (80 mm length × 40 mm width × 4 mm thickness) were welded together using the LTW technique [62], as shown in Figure 1. PC is a transparent, hard, tough, stiff, amorphous thermoplastic with outstanding impact resistance and strength. It has been widely used in the automotive industry, packaging, electronics devices, cash dispenser, electrical devices, baby bottles, and sporting goods. PMMA is a transparent, rigid thermoplastic polymer with outstanding stiffness, strength, and dimensional stability. It has high resistance to weathering and ultraviolet light. The mechanical and thermophysical properties of PC and PMMA are tabulated in Table 1. The welding process was accomplished using an Nd:YVO_4_ laser with a wobbling feature [62]. The beam diameter, wavelength, pulse width, average power, and spot diameter were 1.2 mm, 1064 nm, 4.2 ns, 9.28 W, and 50 µm, respectively. A lap joint with 30 mm overlap is formed by putting a transparent PMMA on a transparent PC plaque. A black marker was used to draw a straight line in the location of the weld line which acted as an absorber to laser light. The stand-off distance and laser spot diameter were considered as constant parameters during all experiments. The lap joint assembly was held on the welding table using screw clamps. These clamps provided the assembly with the required forces for ensuring perfect heat transfer from the black line-welded plaques. 

Laser power (7.89, 8.12, and 8.35 W), welding speed (2, 3, and 4 mm/s), pulse frequency (25, 30, and 35 kHz), wobble frequency (1, 3, and 5 kHz), and wobble width (0.4, 0.6 and 0.8 mm) were considered as the control factors of the welding process; whereas, seam width (SW) and shear strength (WS) of the weld was considered as the process responses [62]. The shear test was carried out on the universal testing machine Instron^®^ model 8801. To ensure dominant shear loading over bending loading, two square plaques (40 mm × 40 mm) were attached to the free ends of PMMA and PC plaques using super glue. Moreover, the loading area of all specimens was roughened to prevent the possibility of slipping during the test. The joint strength was considered as the maximum load at the failure of the joint during the test. The SW was measured using an OLYMPUS 3-D optical microscope model STM-6. All measurements were carried out three times to minimize measurement errors, and the average of the three readings was used as the final response. A face-centered composite design was employed to design the experiments considering three levels of each process control factor. In this design, center points, star points, and factorial points were 8, 10, and 32, respectively. According to the established design plan, fifty experiments were carried out as tabulated in Table 2. 

## 3. Modeling Approach

In this section, the modeling approach is discussed. The basics and mathematical formulation of MLP and AO are introduced. Then, the hybrid AO-MLP developed to predict the shear strength and seam width of the welded joints is presented.

### 3.1. Multilayer Perceptron (MLP)

A multilayer perceptron (MLP) is a well-known feedforward ANN with a fully connected structure. It is composed of multiple layers containing nodes (perceptrons) that act as data processing units. A typical MLP model has one input layer, one or multiple hidden layers, and one output layer. All these layers contain many neurons depending on the modeled process. Each neuron, except the input ones, has a nonlinear transfer function. This type of ANN model is trained using a supervised learning procedure called the backpropagation technique. The input neurons receive the input data, and all input and/or output neurons are connected to the preceding and following neurons in other adjacent layers. For each hidden neuron, given n inputs from the input layer, the input value $x_{i}^{r}$ of the r  layer is computed as:

(1) $$x_{i}^{r}=\Phi \left(\sum_{j=1}^{n}z_{i}^{r-1}\times G_{ji}^{r}+S_{i}^{j}\right)$$

where $z_{i}^{r-1}$ denotes the preceding layer output; $G_{ji}^{r}$ denotes the weight connecting to the neuron to the j-th input; $S_{i}^{j}$ denotes the bias value of i-th neuron; $\Phi \left(.\right)$ denotes the transfer function. The backpropagation process is executed at the output layer by employing the following loss function:

(2) $$L=L\left(z_{1}^{r},\ldots ,z_{h}^{r}\right)=\sum_{i=1}^{h}{\left(z_{i}^{r}-e_{i}\right)}^{2}$$

where the r-th and $e_{i}\;$ denote the output layer and the expected output of the i-th output neuron. The weights of the model are updated using many techniques such as gradient descent, mini-batch gradient descent, Nesterov accelerated gradient, and stochastic gradient descent.

In this study, we employed a gradient descent technique as an easy-to-implement computational technique. The weights are updated according to the following formula:

(3) $$G_{jn}^{r}=G_{jn}^{r-1}-µ\times \frac{\partial L}{\partial G_{jn}^{r-1}}$$

where the  µ denotes the rate of the learning process.

### 3.2. Archimedes Optimizer

The AO is a new metaheuristic optimizer developed to solve engineering optimization problems. It is inspired by Archimedes’ theorem which states that the upward force exerted on an immersed body in a certain fluid equals the weight of the displaced fluid [63]. In a typical AO model, several immersed bodies are used as the algorithm’s population. In the beginning, the locations of the bodies are initialized as follows:

(4) $$BL_{k}=LLP_{k}+rand\times \left(ULP_{k}-LLP_{k}\right)$$

where $BL_{i}$ denotes the position of the k-th body,  LLP denotes the lower limit of position decision parameters, ULP denotes the upper limit of position decision parameters, and rand is a generator of random numbers.

After that, the volume and density of all bodies are randomly defined as follows:

(5) $$V_{k}=rand$$

(6) $$D_{k}=rand$$

where $V_{k},D_{k}$ and rand denote the volume of k−th body, the density of k−th body, and a generator of random numbers, respectively.

Then, the acceleration of bodies is determined as follows:

(7) $$f_{i}=LLF_{k}+rand\times \left(ULF_{k}-LLF_{k}\right)\;$$

where $f_{i}$ denotes the acceleration of the k−th body,  LLF denotes the lower limit of acceleration decision parameters, ULF denotes the upper limit of acceleration decision parameters, and rand is a generator of random numbers.

The volume and density of the bodies are updated according to the following formulas:

(8) $$V_{k}^{t+1}=V_{k}^{t}+rand\times \left(V_{b}-V_{k}^{t}\right)$$

(9) $$D_{k}^{t+1}=D_{k}^{t}+rand\times \left(D_{b}-D_{k}^{t}\right)$$

where $V_{k}^{t},V_{k}^{t+1},\;D_{k}^{t},D_{k}^{t+1},\;V_{b}\;$ and $D_{b}$ denote the k−th body’s volume at iteration t, k−th body’s volume at iteration t+1, k−th body’s density at iteration t, k−th body’s density at iteration t+1, best body’s volume, and best body’s density, respectively.

The bodies begin to collide with each other until reaching the equilibrium position after some time. This process is mathematically modeled using a transfer function which is employed to change the search behavior from the exploration stage to the exploitation stage. This transfer function is defined as:

(10) $$tf=exp\left(\frac{g-h}{h}\right)$$

where tf, g, and h denote the transfer function, the number of iterations, and the maximum number of iterations.

Additionally, the AO employs a decreasing density function $\left(df\right)$ to change the search mode from global mode to local mode. This function is defined as follows:

(11) $$df^{t+1}=exp\left(\frac{g-h}{h}\right)-\left(\frac{g}{h}\right)$$

During the exploration stage, bodies collide with each other and df≤0.5. The acceleration of a certain body is updated according to the following formula:

(12) $$f_{k}^{t+1}=\frac{D_{rm}+V_{rm}\times f_{rm}}{D_{k}^{t+1}+V_{k}^{t+1}}$$

where $f_{k}^{t+1}$, $f_{rm},\;V_{rm},\;and\;D_{rm}\;$ denote the k−th body’s acceleration at iteration t+1, body’s acceleration with random material, body’s volume with random material, and body’s density with random material.

During the exploitation phase, bodies do not collide with each other, and df>0.5. The acceleration of a certain body is updated according to the following formula:

(13) $$f_{k}^{t+1}=\frac{D_{b}+V_{b}\times f_{b}}{D_{k}^{t+1}+V_{k}^{t+1}}$$

where $f_{b}$ denote the best body’s acceleration.

The acceleration is normalized using the following formula:

(14) $$f_{knorm}^{t+1}=\sigma \times \frac{f_{k}^{t+1}-f_{min}}{f_{max}-f_{min}}-\upsilon$$

$f_{knorm}^{t+1},\;f_{k}^{t+1},f_{max},\;f_{min},\sigma \;$ and υ denote normalized acceleration, maximum acceleration, minimum acceleration, first normalization parameter, and second normalization parameter, respectively. 

At the beginning of optimization, acceleration has a large numerical value. Then, it decreases during each iteration. This process accelerates reaching the global solution. Finally, the locations of the bodies are updated according to the following formulas:

If df≤0.5,

(15) $$BL_{k}^{t+1}=BL_{k}^{t}+C_{1}\times rand\times f_{knorm}^{t+1}\times df\times \left(BL_{rand}-BL_{k}^{t}\right)$$

If df>0.5,

(16) $$BL_{k}^{t+1}=BL_{b}^{t}+C_{2}\times C_{3}\times tf\times rand\times f_{knorm}^{t+1}\times df\times \left(C_{3}\times tf\times BL_{b}-BL_{k}^{t}\right)$$

where $C_{1},C_{2}\;and\;C_{3}\;$ are updating constants. Moreover, $BL_{k}^{t},\;BL_{k}^{t+1},BL_{b}^{t},\;BL_{b},\;and\;BL_{rand}$ denote the location of k−th body at iteration t, the new location of k−th body at iteration t+1, the best position of a certain body for iteration t, the best position for all iterations, and the random position of bodies.

### 3.3. Optimized Model

The metaheuristic optimizers (PSO and AO) are employed in this study to obtain the optimal values of biases $\left(S_{i}^{j}\right)$ and weights $\left(G_{ji}^{r}\right)\;$ that lead to the highest model accuracy. Biases and weights are treated as AO bodies which are obtained by minimizing the cost function through the optimization process. The employed cost function was a mean square error (*MSE*) function which is given as follows:

(17) $$MSE=\frac{\sum_{a=1}^{NS}\sum_{b=1}^{NM}{\left(target_{ab}-predicted_{ab}\right)}^{2}}{NS}$$

where $predicted_{ab}$ and $target_{ab}\;$ denote the predicted and real values of output b-th for a training sample a-th. NS and NM are the total number of training samples and the total number of outputs, respectively. 

## 4. Results and Discussions

**Figure 2:**
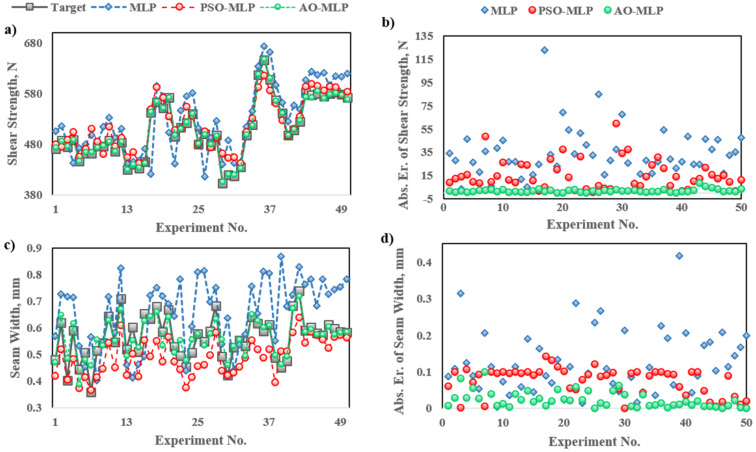
Target and predicted data using MLP, PSO-MLP, and AO-MLP for: (**a**) data of shear strength; (**b**) absolute error of shear strength; (**c**) data of seam width; (**d**) absolute error of seam width.

**Figure 3:**
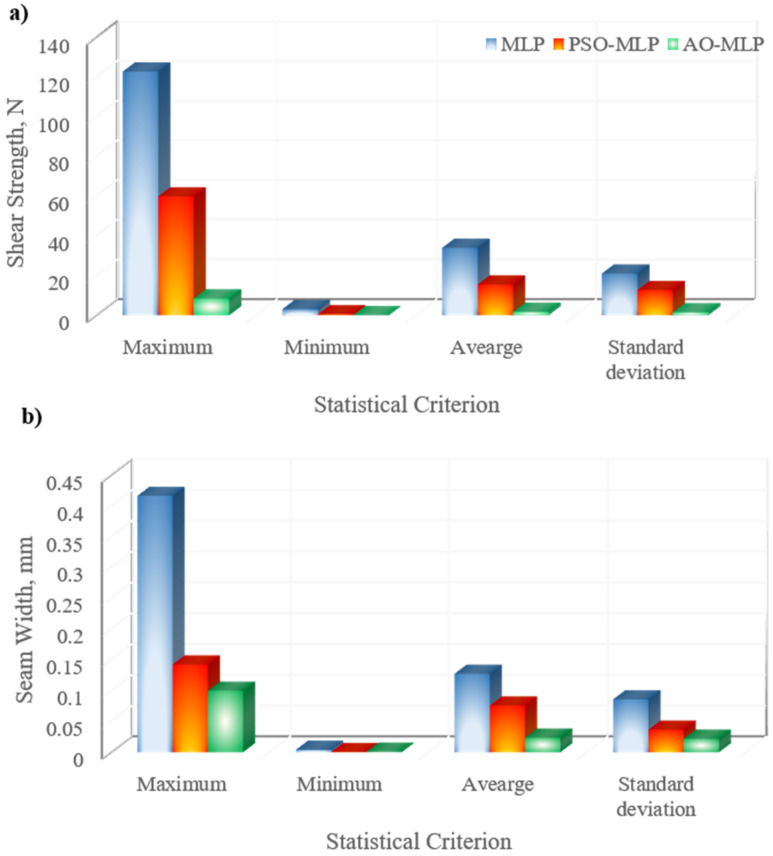
Maximum, minimum, average, and standard deviation of absolute errors between predicted data using MLP, PSO-MLP, and AO-MLP and target ones for: (**a**) shear strength and (**b**) seam width.

The joint characteristics in terms of shear strength and seam width were predicted using three AI models, namely, MLP, PSO-MLP, and AO-MLP. Like other AI tools, models were trained using experimental data to learn and figure out the main features and relationships that exist in the data. Forty datasets were utilized to train the models which represent 80% of the whole datasets. Then, the models were tested using the remaining datasets. Figure 2a shows the predicted shear strength of the welded joints using MLP, PSO-MLP, and AO-MLP and the target data. There is excellent agreement between the target data and those predicted using AO-MLP. A moderate agreement between the target data and those predicted using PSO-MLP is also observed. The worst agreement between the target data and predicted data is observed in the case of a standalone MLP model. The same trend is observed for seam width as shown in Figure 2a. The best agreement is observed for AO-MLP followed by PSO-MLP; whereas, MLP shows the worst agreement. The absolute errors between the predicted shear strength of different samples and the experimental ones reveal that AO-MLP has the minimum error among other models, as shown in Figure 2b. Additionally, the absolute errors between the predicted seam width of different samples and the experimental ones reveal that AO-MLP has the minimum error among other models as shown in Figure 2b. The statistical analysis of absolute errors between predicted data using MLP, PSO-MLP, and AO-MLP and target ones for shear strength and seam width is shown in Figure 3 and listed in Table 3, considering the maximum, minimum, average, and standard deviation of the data. MLP has maximum error values of 122.709 N and 0.416 mm for shear strength and seam width, respectively, which are the highest among all models; as PSO-MLP has 59.834 N and 0.142 mm, whereas AO-MLP has 8.464 N and 0.099 mm. The maximum absolute error of MLP is more than 14 and 4 times that of AO-MLP for shear strength and seam width, respectively, which reveals the tendency of MLP to predict some data with outlier errors that may destroy the prediction process. AO-MLP has minimum error values of 0.026 N and 0.0008 mm for shear strength and seam width, respectively, which are the lowest among all models; as MLP has 3.117 N and 0.004 mm, whereas PSO-MLP has 0.5123 N and 0.0003 mm. The average absolute error and standard deviation of MLP are more than 19 and 13 times that of AO-MLP for shear strength, respectively, whereas they are more than 5 and 3 times that of AO-MLP for seam width. The low values of average shear length and seam width obtained by AO-MLP reveal the better accuracy of AO-MLP compared with MLP and PSO-MLP. The low values of the standard deviation of shear length and seam width obtained by AO-MLP reveal the lower variance of AO-MLP compared with MLP and PSO-MLP. 

**Figure 4:**
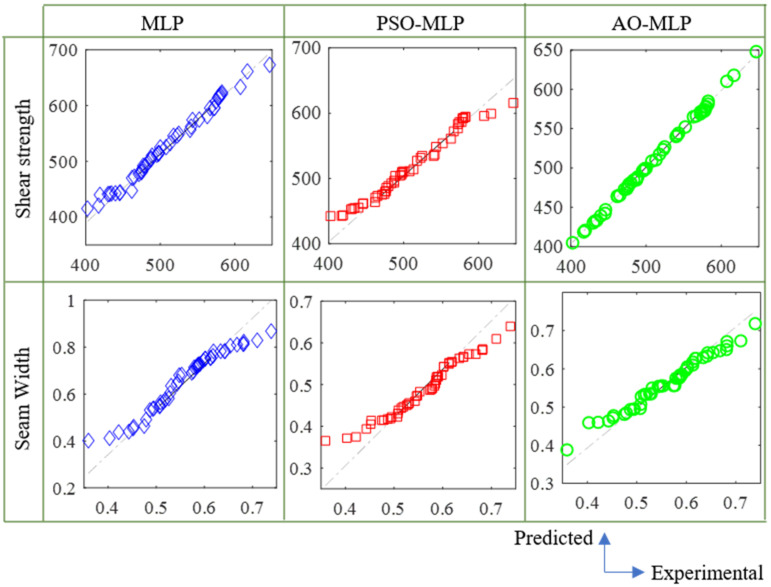
Q-Q plots of predicted data using MLP, PSO-MLP, and AO-MLP and target ones for shear strength and seam width.

**Figure 5:**
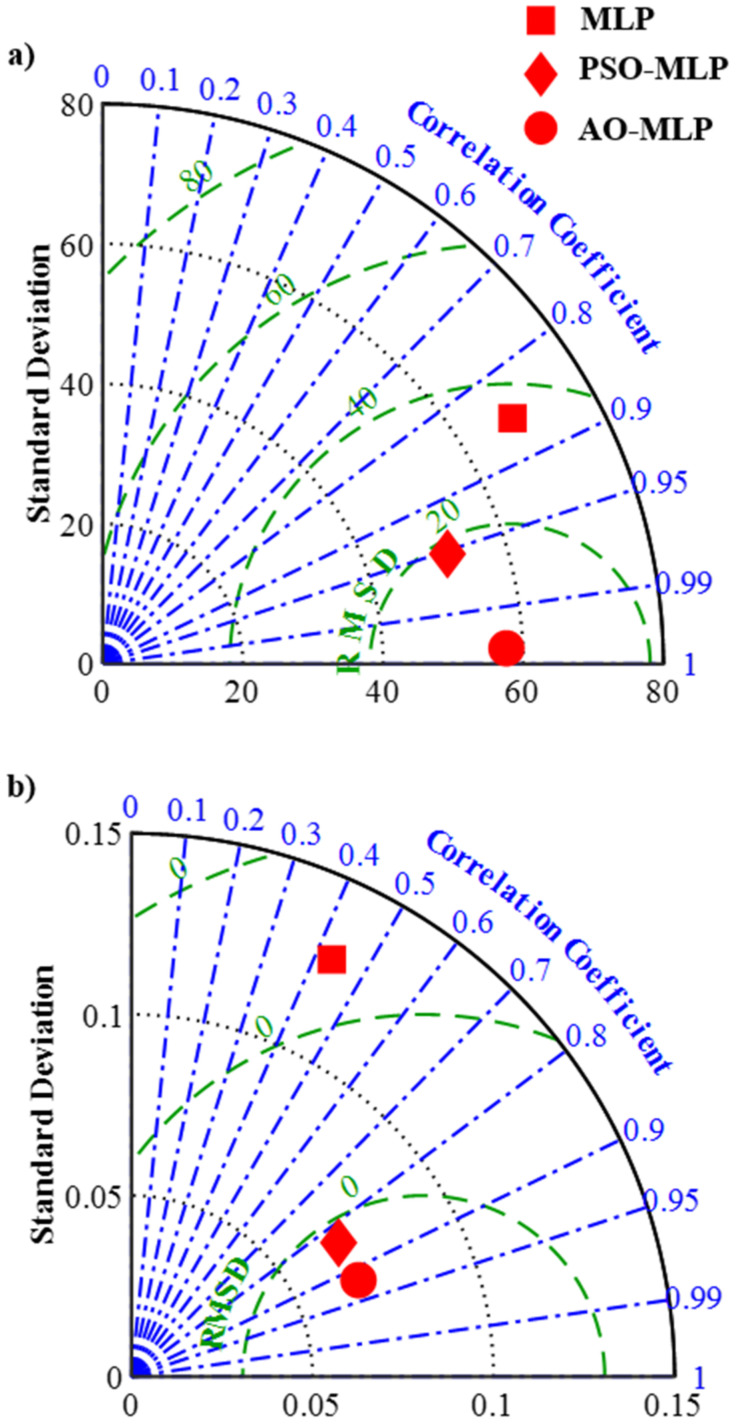
Taylor diagrams of predicted data using MLP, PSO-MLP, and AO-MLP and target ones for: (**a**) shear strength and (**b**) seam width.

Q-Q plots shown in Figure 4 confirm the outperformance of AO-MLP over MLP and PSO-MLP. The plotted points of AO-MLP (green points) have a lower offset from the straight line compared with that of MLP (blue points) and PSO-MLP (red points). The low offsets between the plotted points and the straight line indicate the high correlation between the predicted and target data, and consequently, the high accuracy of the model. It is observed from this figure that AO-MLP has the highest accuracy followed by PSO-MLP, whereas MLP has the lowest accuracy. Taylor diagrams shown in Figure 5 also confirm the outperformance of AO-MLP over MLP and PSO-MLP. AO-MLP has the highest correlation coefficient, lowest root-mean-square deviation, and reasonable standard deviation compared with that of MLP and PSO-MLP. In the case of shear strength, correlation coefficients are (0.857, 0.952, and 0.999), the root-mean-square deviation is (35.058, 18.073, and 2.283), and the standard deviation is (68.184, 51.626, and 57.639) for MLP, PSO-MLP, and AO-MLP, respectively. In the case of seam width, correlation coefficients are (0.433, 0.839, and 0.920), the root-mean-square deviation is (0.118, 0.044, and 0.032), and the standard deviation is (0.1280, 0.068 and 0.068) for MLP, PSO-MLP, and AO-MLP, respectively. 

**Figure 6:**
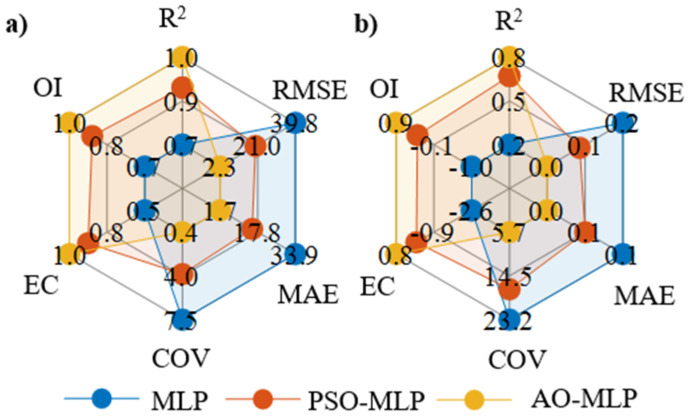
Radar diagrams of different statistical measures used to evaluate the performance MLP, PSO-MLP, and AO-MLP for: (**a**) shear strength and (**b**) seam width.

The accuracy of the models are assessed using six of the most commonly used statistical measures, namely, coefficient of determination (R^2^), root means square error (RMSE), mean absolute error (MAE), coefficient of variance (COV), efficiency coefficient (EC), and coefficient of residual mass (CRM) and overall index (OI) [64]. The model with the highest accuracy should have unity values of R^2^, EC, and OI. On the other hand, the model with the highest accuracy should have minimum values of RMSE, MAE, and COV. As shown in Figure 6 and tabulated in Table 4, AO-MLP outperformed MLP and PSO-MLP models. AO-MLP has the highest values of R^2^, EC, and OI of (0.998, 0.998, and 0.994) and (0.847, 0.841, and 0.878) for shear strength and seam width, respectively. These values are much higher than that of MLP and PSO-MLP. In the case of MLP, they are (0.735, 0.531, and 0.684) and (0.187, −2.602, and −1.002) for shear strength and seam width, respectively. In the case of PSO-MLP, they are (0.907, 0.882, and 0.901) and (0.705, −0.084, and 0.347) for shear strength and seam width, respectively. AO-MLP has the lowest values of RMSE, MAE, and COV of (2.283, 1.729, and 0.447) and (0.0321, 0.023, and 5.708) for shear strength and seam width, respectively. These values are much higher than that of MLP and PSO-MLP. In the case of MLP, they are (39.798, 33.925, and 7.523) and (0.153, 0.127, and 23.198) for shear strength and seam width, respectively. In the case of PSO-MLP, they are (19.909, 15.462, and 3.839) and (0.084, 0.075, and 17.117) for shear strength and seam width, respectively. Thus, AO-MLP has better accuracy to predict the shear strength and seam width of the welded joints compared with MLP and PSO-MLP models. This is due to the vital role of AO to obtain the internal parameters of MLP that maximize the model accuracy. Consequently, AO-MLP is recommended to model the LTW process of dissimilar lap joints made of PMMA and PC. 

## 5. Conclusions

In this study, predictive models were developed to model laser welding of dissimilar polymeric joints using the transmission welding technique based on the experimental data reported in [62]. The welded joints were made of polymethyl methacrylate and polycarbonate sheets. The welding process was accomplished using an Nd:YVO_4_ laser with a wobbling feature. A black marker was used to draw a straight line in the location of the weld line which acted as an absorber to laser light. Laser power, welding speed, pulse frequency, wobble frequency, and wobble width were considered as the control factors of the welding process; whereas, seam width and shear strength of the weld was considered as the process responses. A face-centered composite design was employed to design the experiments considering three levels of each process control factor. The models were composed of conventional multi-layer perceptrons coupled with the Archimedes optimizer or particle swarm optimizer. The prediction accuracy of all models was compared using different error measures. Archimedes’ optimizer model outperformed the pure model and particle swarm model. It has the highest values of coefficient of determination of 0.998 and 0.847 for shear strength and seam width, respectively. These values are much higher than that of the pure model and particle swarm model, which reveals its ability to predict the process responses with excellent accuracy. For future work, it is recommended to employ other artificial intelligence tools as well as metaheuristic optimizers to model and optimize laser welding processes. 

## Figures and Tables

**Table 1:** Mechanical and thermophysical properties of PC and PMMA.

Property	Thermal Conductivity at 25 °C (W/mK)	Specific Heat (J/(kg °C))	Density (kg/m^3^)	Glass Transition Temperature (°C)	Linear Thermal Expansion (×10^−5^/k)	Poisson’s Ratio	Young’s Modulus (MPa)	Luminance Transmission
PC	0.20	1100	1160	141	6.5	0.399	2400	89%
PMMA	0.21	1270	1190	83	6.3	0.328	3300	92%

**Table 2:** Experimental plan [62].

	Laser Power (W)	Welding Speed (mm/s)	Pulse Frequency (kHz)	Wobble Frequency (kHz)	Wobble Width (mm)	Seam Width (mm)	Shear Strength (N)
1	7.89	2	25	1	0.4	0.479	471.24
2	8.35	2	25	1	0.4	0.619	487.23
3	7.89	2	35	1	0.4	0.403	474.38
4	8.35	2	35	1	0.4	0.589	488.87
5	7.89	4	25	1	0.4	0.443	446.08
6	8.35	4	25	1	0.4	0.507	462.18
7	7.89	4	35	1	0.4	0.359	461.72
8	8.35	4	35	1	0.4	0.515	476.22
9	7.89	2	25	1	0.8	0.543	475.44
10	8.35	2	25	1	0.8	0.643	487.43
11	7.89	2	35	1	0.8	0.546	465.08
12	8.35	2	35	1	0.8	0.71	483.57
13	7.89	4	25	1	0.8	0.519	429.78
14	8.35	4	25	1	0.8	0.602	439.77
15	7.89	4	35	1	0.8	0.509	431.92
16	8.35	4	35	1	0.8	0.654	445.41
17	7.89	2	25	5	0.4	0.634	543.24
18	8.35	2	25	5	0.4	0.681	562.98
19	7.89	2	35	5	0.4	0.586	552.13
20	8.35	2	35	5	0.4	0.668	572.37
21	7.89	4	25	5	0.4	0.529	495.33
22	8.35	4	25	5	0.4	0.495	513.08
23	7.89	4	35	5	0.4	0.453	522.72
24	8.35	4	35	5	0.4	0.508	539.97
25	7.89	2	25	5	0.8	0.577	479.69
26	8.35	2	25	5	0.8	0.548	500
27	7.89	2	35	5	0.8	0.587	477.03
28	8.35	2	35	5	0.8	0.682	497.32
29	7.89	4	25	5	0.8	0.493	402.28
30	8.35	4	25	5	0.8	0.422	419.03
31	7.89	4	35	5	0.8	0.526	417.17
32	8.35	4	35	5	0.8	0.553	434.41
33	7.89	3	30	3	0.6	0.531	497.65
34	8.35	3	30	3	0.6	0.642	517.77
35	8.12	3	25	3	0.6	0.618	616.82
36	8.12	3	35	3	0.6	0.587	646.59
37	8.12	2	30	3	0.6	0.613	607.47
38	8.12	4	30	3	0.6	0.487	566.94
39	8.12	3	30	3	0.4	0.452	541.29
40	8.12	3	30	3	0.8	0.475	498.12
41	8.12	3	30	1	0.6	0.682	507.71
42	8.12	3	30	5	0.6	0.739	524.71
43	8.12	3	30	3	0.6	0.591	582.43
44	8.12	3	30	3	0.6	0.602	577.43
45	8.12	3	30	3	0.6	0.579	580.12
46	8.12	3	30	3	0.6	0.574	574.43
47	8.12	3	30	3	0.6	0.613	579.23
48	8.12	3	30	3	0.6	0.598	582.58
49	8.12	3	30	3	0.6	0.585	578.57
50	8.12	3	30	3	0.6	0.583	572.41

**Table 3:** Statistical analysis of absolute errors between predicted data using MLP, PSO-MLP, and AO-MLP and target ones.

Statistical Criteria	Shear Strength, N			Seam Width, mm		
	MLP	PSO-MLP	AO-MLP	MLP	PSO-MLP	AO-MLP
Maximum	122.709	59.834	8.464	0.416	0.142	0.099
Minimum	3.117	0.512	0.026	0.004	0.0003	0.0008
Average	33.925	15.462	1.729	0.127	0.075	0.024
Standard deviation	21.018	12.669	1.505	0.085	0.036	0.022

**Table 4:** Different statistical measures used to evaluate the performance MLP, PSO-MLP, and AO-MLP.

		**R^2^**	**RMSE**	**MAE**	**COV**	**EC**	**OI**
Shear strength	MLP	0.735	39.798	33.925	7.523	0.531	0.684
	PSO-MLP	0.907	19.909	15.462	3.839	0.882	0.901
	AO-MLP	0.998	2.283	1.729	0.447	0.998	0.994
Seam Width	MLP	0.187	0.153	0.127	23.198	−2.602	−1.002
	PSO-MLP	0.705	0.084	0.075	17.117	−0.084	0.347
	AO-MLP	0.847	0.0321	0.023	5.708	0.841	0.878
